# Circumcision status at HIV infection is not associated with plasma viral load in men: analysis of specimens from a randomized controlled trial

**DOI:** 10.1186/s12879-018-3257-8

**Published:** 2018-07-28

**Authors:** Stephanie M. Davis, Sherri Pals, Chunfu Yang, Elijah Odoyo-June, Joy Chang, Maroya Spalding Walters, Walter Jaoko, Naomi Bock, Larry Westerman, Carlos Toledo, Robert C. Bailey

**Affiliations:** 1 Division of Global HIV/AIDS and Tuberculosis, US Centers for Disease Control, Atlanta, GA USA; 2 Division of Global HIV/AIDS and Tuberculosis, US Centers for Disease Control, Kisumu, Kenya; 3 Department of Medical Microbiology, University of Nairobi, Nairobi, Kenya; 4 Division of Epidemiology and Biostatistics, University of Illinois Chicago School of Public Health, Chicago, IL USA; 5 Division of Global HIV and TB, HIV Prevention Branch US Centers for Disease Control and Prevention, 1600 Clifton Rd. NE Mail Stop E-04, Atlanta, GA 30329 USA

**Keywords:** Circumcision, HIV prevention, Viral load, Clinical trials, Intervention

## Abstract

**Background:**

Male circumcision provides men with approximately 60% protection from acquiring HIV infection via heterosexual sex, and has become a key component of HIV prevention efforts in sub-Saharan Africa. Possible mechanisms for this protection include removal of the inflammatory anaerobic sub-preputial environment and the high concentration of Langerhans cells on the inside of the foreskin, both believed to promote local vulnerability to HIV infection. In people who do acquire HIV, viral load is partially determined by infecting partner viral load, potentially mediated by size of infecting inoculum. By removing a portal for virion entry, prior male circumcision could decrease infecting inoculum and thus viral load in men who become HIV-infected, conferring the known associated benefits of slower progression to disease and decreased infectiousness.

**Methods:**

We performed an as-treated analysis of plasma samples collected under a randomized controlled trial of male circumcision for HIV prevention, comparing men based on their circumcision status at the time of HIV acquisition, to determine whether circumcision is associated with lower viral load. Eligible men were seroconverters who had at least one plasma sample available drawn at least 6 months after infection, reported no potential exposures other than vaginal sex and, for those who were circumcised, were infected more than 6 weeks after circumcision, to eliminate the open wound as a confounder. Initial viral load testing indicated that quality of pre-2007 samples might have been compromised during storage and they were excluded, as were those with undetectable or unquantifiable results. Log viral loads were compared between groups using univariable and multivariable linear regression, adjusting for sample age and sexually transmitted infection diagnosis with 3.5 months of seroconversion, with a random effect for intra-individual clustering for samples from the same man. A per-protocol analysis was also performed.

**Results:**

There were no viral load differences between men who were circumcised and uncircumcised at the time of HIV infection (means 4.00 and 4.03 log_10_ copies/mL respectively, *p* = .88) in any analysis.

**Conclusion:**

Circumcision status at the time of HIV infection does not affect viral load in men.

**Trial registration:**

The original RCT which provided the samples was ClinicalTrials.gov trial NCT00059371.

## Background

Male circumcision (MC) has been shown in randomized controlled trials (RCTs) to confer approximately 60% protection to men from acquiring HIV infection via heterosexual sex [1–3]. It has become a key component of HIV prevention efforts in sub-Saharan Africa, with nearly 15 million voluntary medical male circumcisions (VMMC) performed since 2008 [4]. Widely-accepted possible mechanisms for the protective effect include: the removal of the inner foreskin’s high concentration of Langerhans cells, through which HIV can access the immune system; the foreskin’s vulnerability to microabrasions; the elimination of the anaerobic sub-preputial environment that supports a pro-inflammatory microbiome; and the greater incidence of genital ulcer disease (GUD) among uncircumcised men [5].

Nevertheless, many circumcised men will ultimately become HIV-infected. Determinants of plasma viral load (VL) set point among infected individuals are not entirely understood, but in addition to well-established associations like host immunogenetics [6] and viral genotype [7], it is associated with VL in “source” partners [8–10]. Although this relationship is sometimes attributed to viral genotype also, another suggested mechanism is that high source partner VL results in a higher infecting inoculum, which then results in higher VL in the newly-infected partner [11, 12]. (The majority of HIV infections resulting from heterosexual sex are believed to result from a single virion [9, 13], so the effect of inoculum size on VL is not hypothesized to result directly from a higher initial virion population. Instead, a larger inoculum may lead to increased host T-cell activation, which in turn enhances replication of the ultimately ‘successful’ virion [10]). Supporting evidence for inoculum size as an independent determinant of VL includes the association between source partner genital tract factors which would affect magnitude of viral shedding, such as bacterial vaginosis and genital herpes suppression, and VLs in seroconverting partners [12].

**Fig. 1:**
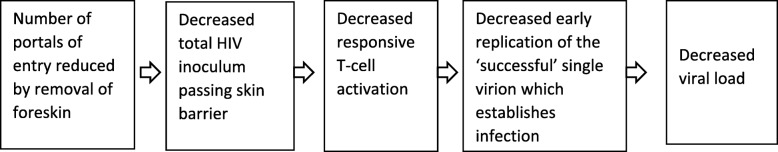
Potential relationship between circumcision status at HIV infection and viral load

If source inoculum affects VL, the same mechanisms that confer partial protection against infection could operate in circumcised men who do become infected to decrease the proportion of virions breaching their skin, lowering inoculum and thus VL. Fig. 1 illustrates this proposed causal chain. If present, such an effect could confer substantial population-level benefit, as VL is a major determinant of both progression to clinical disease and infectiousness [14].

Evidence for the plausibility of this result comes from one study that found MC in genotype-confirmed male source partners was associated with lower HIV VLs among the female partners they infected [10, 15] (adjusted mean difference = −.63 log_10_ copies/mL, *p* = .03). The authors hypothesized the above mechanism in reverse: lower innocula transferred to female partners due to the removal of the male’s infectious foreskin Langerhans cells via MC. This study did not find evidence of lower VL set points associated with MC among men infected by female source partners, but this outcome was limited by small sample size (*N* = 55) and the fact that participants were not randomized with respect to circumcision status.

We used stored plasma samples from the original RCT of MC for HIV prevention conducted in Kisumu, Kenya [2], to investigate whether MC status of male participants who became HIV-infected was associated with their VL.

## Methods

### Enrollment, randomization, sample collection and follow-up in the Kisumu-based RCT

Detailed methods of the RCT are described elsewhere [2] (ClinicalTrials.gov, NCT00059371). Briefly, it was conducted in Kisumu district, western Kenya. Male participants were recruited via newspapers, radio, fliers and street shows and enrolled between 2002 and 2005. Inclusion criteria included being an uncircumcised, HIV-negative, sexually active, 18–24-year-old Kisumu resident. Exclusion criteria included contraindications to, and absolute indications for, surgical MC. An opaque envelope system was used to produce 1:1 allocation between arms. Plasma HIV testing was performed and self-reported HIV exposure data collected at 1, 3, and 6 months after randomization, then at 6-month intervals. The trial was stopped early in December 2006 due to efficacy. Free MC was then offered to all interested participants throughout the follow-up period, causing crossover from the control group. Semi-annual follow-up visits continued through September 2010. The dataset is publically available [16].

### HIV testing and determination of seroconversion and infection dates

Two rapid tests were used at each visit, Determine HIV 1/2 (Abbott Diagnostic Division, Hoofddorp, Netherlands) and Unigold Recombigen (Trinity Biotech, Wicklow, Ireland). If one or both was positive, serum was sent for double enzyme-linked immunosorbent assay testing (Detect HIV 1/2, Adaltis Inc., Montreal, Canada and Recombigen HIV 1/2, Trinity Biotech, Wicklow, Ireland). If at least one ELISA was positive, final confirmatory testing was done by line immunoassay (INNO-LIA HIV 1/2, Immunogenetics NV, Ghent, Belgium). For participants confirmed as HIV positive, the first visit with at least one positive rapid test was designated the HIV seroconversion visit. We calculated the presumed infection date as the date midway between the final negative test and the HIV seroconversion visit.

### Other data

Additional data collected at enrollment and in follow-up included MC procedure date, behavioral and other HIV risk factors, and sexually transmitted infection (STI) diagnoses. High-risk sexual behavior within the 6 months before a visit was defined as self-report of > 2 female sex partners, exchanging gifts or money for sex, or using a condom “less than half the time”.

STI testing included serum rapid plasma reagin (RPR) (Becton Dickinson) with *T. pallidum* particle agglutination (TPHA) confirmation for syphilis and HSV-2 serology; and urine polymerase chain reaction (PCR) for *N. gonorrhoeae* and *C. trachomatis*. Men with urethral discharge also had urethral swabs for *N. gonorrhoeae* culture and PCR, *C. trachomatis* PCR (Roche Diagnostics), and *T. vaginalis* culture (In pouch test). Any genital ulcers were swabbed for *H. ducreyi* culture, and PCR and HSV-2 PCR testing using the Roche Multiplex PCR system. Men were defined as having an STI if any tests were positive except a positive RPR with negative TPHA. The study did not collect data on antiretroviral therapy (ART) initiation among participants; between 2005 and 2011, the Kenyan National HIV Treatment guidelines set a CD4 threshold for ART of <=200/mm^3^.

### Inclusion and exclusion criteria for HIV VL analysis

Individual inclusion criteria for this analysis were: seroconversion during the trial or follow-up; and having at least one available plasma sample drawn at least 6 months after infection date and, for those who became circumcised after infection, drawn 6 months after MC (to eliminate bias from the procedure causing transient VL increases). Individual exclusion criteria were: potential HIV exposures other than vaginal sex at any time (sex with men, intravenous drug use, or blood transfusion); a still-unhealed MC wound less than 3 weeks prior to infection; and for circumcised men, infection date fewer than 6 weeks after MC (unhealed wounds may have facilitated infection among men who resumed sex earlier).

Sample exclusion criteria were also applied. Samples drawn before Jan 1, 2007 were excluded from the final analysis because all but five (of 85) had undetectable or unquantifiable VLs, correlations described below between sample age and VL suggested older samples were subject to degradation, and none of these samples had second aliquots available for quality control. Finally, samples with undetectable or unquantifiable results were also ultimately excluded (see *Data Analysis* section for methods and reasoning). Among eligible men, the first eligible samples, up to three, were used.

### Sample size

The anticipated sample size based on total available aliquots was 123 men (82 circumcised and 41 uncircumcised), which provided 80% power to detect a between-group difference in mean VL of .54 standard deviations, equivalent to .93 log_10_ copies/mL [12]. Power calculations were done using SAS 9.3 PROC POWER TWOSAMPLEMEANS with a two-sample t-test.

### Sample storage and testing

Plasma samples were not tested at the time of collection, and were not thawed at any time before testing. They were stored at − 80 °C, initially at the Kenya Medical Research Institute in Kisumu, and permanently at the Chicago Developmental Center for AIDS Research. VL testing was performed by the International Laboratory Branch of the US Centers for Disease Control’s Division of Global HIV and Tuberculosis in Atlanta, Georgia, using the Abbott HIV-1 Real Time HIV-1 assay on the Abbott m2000 instrument, with a lower limit of detection of 150 copies/mL with a 200 μL plasma input volume. All samples with an available second aliquot were retested and results compared against initial values, with a difference threshold of ≤0.5 log_10_ copies/mL for concurrence.

### Data analysis

In the primary (as-treated) analysis, men were classified as circumcised or uncircumcised based on whether they had been circumcised through the RCT’s services before infection. The secondary (per-protocol) analysis restricted the comparison to men remaining in their original randomization group. Analysis was performed using SAS 9.3 on log_10_-transformed VL results from the first round of testing.

Once a high proportion of samples with undetectable or unquantifiable (VL detected at a concentration below the quantifiability threshold, 2.18 log copies/mL) results was identified, methods for handling these values were devised. Prior to the second round of testing, it was pre-specified that all such samples would be retested if a second aliquot was available, and the values would be included in the analysis if at least 80% of their retested samples were again either undetectable or unquantifiable, increasing confidence that these represented true plasma values rather than degradation of the aliquots.

To compare characteristics between groups, we used Fisher’s exact Chi-square test for categorical variables, and t-tests for continuous variables. Pearson correlation coefficients were used to examine the relationship between sample age and log VL.

PROC MIXED was used to test for differences in log VL between groups, with a random effect for individual to account for the correlation between samples from the same man, This approach yields results similar to an analysis on log VL means computed for each man and then compared between groups, but also allows for the use of sample-level covariates such as sample age.

## Results

Specimen collection dates for included samples ranged from January 10, 2007 to September 30, 2010. VL results were concurrent between aliquots for all retested quantifiable samples, but samples with initially undetectable or unquantifiable VL results did not meet the prespecified criteria and were excluded, leaving 124 included samples representing 63 seroconverting participants (37 circumcised and 26 uncircumcised). Twenty-three men had only one sample. With the reduction in size, the analyzed sample had 80% power to detect a between-group difference of .73 standard deviations, or 1.26 log_10_ copies/mL. Log viral load results had a range of 2.33 to 6.21, with a median of 4.04 (IQR 3.57–4.53). For men with two or three results, the intraclass correlation between log viral loads was strong at 0.63. Table 1 compares key demographic and risk characteristics, and sample-level characteristics of draw timing and age at testing, for circumcised and uncircumcised participants. Groups were similar demographically. Circumcised men had a nonsignificant higher prevalence of risky behavior and lower prevalence of STI. Samples were similar in draw timing and age at testing.

**Table 1:** Characteristics of circumcised and uncircumcised HIV-1 seroconverters and their samples (as-treated analysis)

Characteristic	Circumcised mean (SD) or % (n)	Uncircumcised mean (SD) or % (n)	*p*-value^a^
Individual-level (*N* = 37 circumcised, 26 uncircumcised)			
Mean age (years)	20.0 (1.5)	20.4 (1.6)	.32
Mean educational attainment (years)	9.3 (2.1)	10.0 (2.2)	.20
STI diagnosis within 3.5 months of seroconversion - % (n)	13.5 (5)	34.6 (9)	.08
High-risk sex within 6 months of seroconversion - % (n)	70.3 (26)	53.8 (14)	.20
Sample-level (*N* = 72 circumcised, 52 uncircumcised)			
Mean (days) between seroconversion and draw date	516.2 (210.7)	558.8 (330.3)	.42
Percent (n) drawn < 12 mo. post seroconversion	26.4 (19)	34.6 (18)	.28
Percent (n) drawn 12–17 mo. post seroconversion	31.9 (23)	23.1 (12)	
Percent (n) drawn > 18 mo. post seroconversion	27.8 (20)	19.2 (10)	
Percent (n) drawn > 24 mo. post seroconversion	13.9 (10)	23.1 (12)	
Mean age (days) of sample(s) at testing	2646.5 (367.5)	2702.4 (375.5)	.41

^a^Tests for continuous variables were done using t-tests; tests for categorical variables were done using exact Chi-square tests

**Fig. 2:**
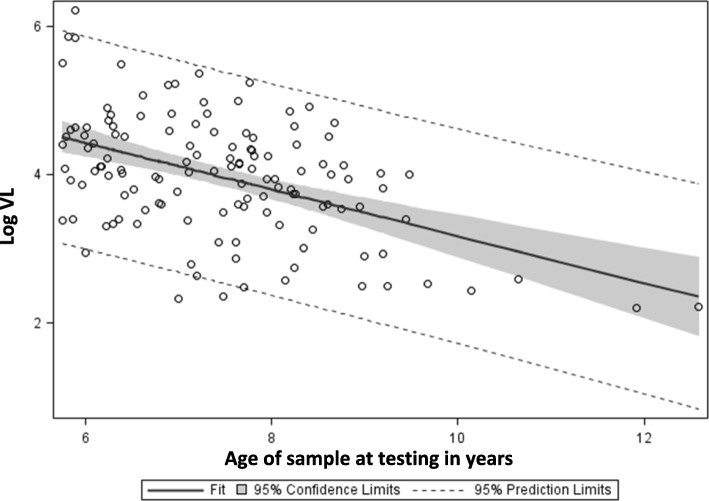
Relationship between age of sample and log_10_ viral load

**Fig. 3:**
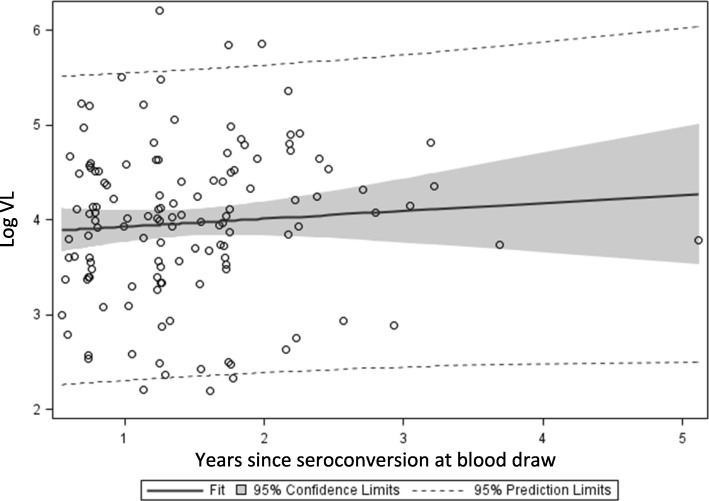
Relationship between time since seroconversion and Log_10_ viral load

A strong linear correlation was found between VL and sample age (Fig. 2; *r* = −.48, *p* < .0001, R^2^ = .23); a quadratic term was tested for significance to assess for a nonlinear association, but was not significant and was therefore dropped. No significant correlation was found between VL and duration of infection at time of blood draw (Fig. 3; *r* = .07, *p* = 0.40).

No significant difference in log VL was found between circumcised and uncircumcised men in the as-treated (means 4.00 and 4.03 log copies/mL respectively, *p* = .88) or per-protocol (means 4.06 and 3.99 log copies/mL, *p* = .76) analyses. This remained true after adjusting for STI diagnosis within 3.5 months of seroconversion and age of sample, for both the as-treated (means 3.96 and 4.09 log copies/mL, *p* = .47) and the per-protocol (means 3.99 and 4.04 log copies/mL, *p* = .80) analysis.

## Discussion

Our results do not support an effect of circumcision status larger than 1.26 log_10_ copies/mL at the time of seroconversion on VL after 6 months. This does not rule out a clinically significant effect: a 1 log difference in VL was found in Uganda to be associated with an adjusted hazard ratio of 3.09 for death and 2.75 for progression to AIDS [17] and the 1 log difference between a VL of 10,000 and 100,000 copies/mL has been modeled to be associated with approximately a 5-year difference in asymptomatic survival [18]. However, this finding is consistent with the observational results reported by Lingappa et al. in participants from southern and eastern African countries, and represents gold-standard data due to randomization which is unlikely to become available from future studies. It also helps establish the assumptions needed for sample size planning for any observational studies further examining the issue. Given the mechanistic biological nature of the proposed effect, results are also likely to be valid across populations.

If the lack of observed effect reflects biological fact, the explanation may lie in the set of portals of entry available to HIV virions on the male genitalia. In addition to the foreskin, the glans, urethral mucosa and possibly fossa navicularis are all portals across which translocation of cell-associated HIV-1 has been demonstrated in vivo [19]. Given the low per-act probability of female-to-male transmission through *any* of these portals (4 per 10,000 infectious exposures in a recent systematic review) [20], it seems to follow that the probability of transmission through any single portal is even lower, and thus that in any one act leading to infection, the most likely scenario is that only one portal is involved. Thus circumcision would remove one portal, decreasing infection risk, but would not affect the size of an inoculum passing through a different portal, thus not affecting VL if infectious becomes established. Others have found higher per-act transmission probabilities such as 0.38% in developing countries in the absence of commercial sex work or genital ulceration, [21] but also at this higher magnitude, a single portal seems likely to be involved in the majority of transmissions.

Conversely, the previously-observed lower VLs in women with circumcised male source partners are more likely to be related to total male viral shedding, which would be expected to come from multiple “portals of exit” during sex, including semen and penile mucosa. Shedding has been shown via coronal lavage to be reduced in HIV-infected men after healing from MC, compared to pre-MC levels [22].

It is also possible that infecting inoculum does not actually impact VL, though this would not explain Lingappa et al.’s [12] observed association between male infecting partner circumcision and lower female partner VL in their baseline data.

Finally, it is possible that there is a population-level effect of circumcision status at time of infection on men’s VL, but that it is mediated by the known protective associations of MC with risk for genital ulcer disease (GUD) [23], thus decreasing VL indirectly through the same mechanisms postulated for a direct effect. Lingappa et al. [12] did not find an association between HSV-2 serology and VL set point, but thoroughly testing this possibility would require a much larger sample of circumcised and uncircumcised seroconverters with sufficient prevalence of GUD to estimate a true effect.

The major potential confounder is the effect of sample age on VL. Although controlling for this did not impact results, the effect of storage time on VL can have an unpredictable [24] component, which could have caused residual confounding. However, if substantial, this component should have eroded all observed associations; the observed correlations between VL and sample age, and between VLs from the same participant, should not have been seen.

Another potential confounder is ART initiation, which was not captured in the study data. However, between 2005 and 2011, Kenyan National HIV Treatment guidelines set a CD4 threshold for ART of <=200/mm^3^ in the absence of clinical indications, and the maximum elapsed time of 5.1 years between calculated infection date and blood draw in our sample makes it unlikely that participants would have progressed to meet this criterion and start treatment. If any did, elimination of samples with undetectable viral load would also have been likely to exclude them. In other respects, although circumcised participants were somewhat more likely to report risky behavior, the difference was not significant, and VLs would not necessarily be higher in infections contracted through high-risk sex.

Finally, for participants with only one sample, stability of VL over time cannot be demonstrated; therefore, VL cannot necessarily be considered equivalent to VL set point.

## Conclusions

Though circumcision in HIV-positive men has an observed association with HIV VL in female partners infected by them, this study provides no evidence that circumcision status at HIV seroconversion affects VL after 6 months in men.

## Abbreviations

ART: Antiretroviral therapy
MC: Male circumcision
RCT: Randomized controlled trial
RPR: Rapid plasma reagin
STI: Sexually transmitted infection
TPHA: *T. pallidum* particle agglutination
VL: Viral load
VMMC: Voluntary medical male circumcision
